# Supplementary material for: Practice Patterns of Antithrombotic Therapy during the Early Postoperative Course of Cardiac Surgery
Source: J Clin Med. 2023 Mar 3;12(5):2029. doi: 10.3390/jcm12052029 (PMC10004004; doi:10.3390/jcm12052029)
Supplement: Supplementary file 1 [file jcm-12-02029-s001.zip › jcm-2165222-supplementary.pdf]

Journal of Clinical Medicine

Supplementary materials

Practice Patterns of Antithrombotic Therapy during the Early Postoperative Course of Cardiac Surgery

Thomas Klein [1,2], Hugo Bignolas [1], Nicolas Mongardon [3,4,5], Osama Abou-Arab [6], Pierre Grégoire Guinot [7,8], Adrien Bouglé [9,10] and Philippe Guerci [1,2,*]

[1] Department of Anesthesiology and Critical Care Medicine, Cardiothoracic and Vascular Anesthesia, University Hospital of Nancy-Brabois, 54511 Vandoeuvre-lès-Nancy, France
[2] INSERM 1116, DCAC, University of Lorraine, 54500 Vandoeuvre-lès-Nancy, France
[3] Service d'Anesthésie-Réanimation Chirurgicale, DMU CARE, DHU A-TVB, Assistance Publique-Hôpitaux de Paris (AP-HP), Hôpitaux Universitaires Henri Mondor, 94010 Créteil, France
[4] U955-IMRB, Equipe 03 "Pharmacologie et Technologies pour les Maladies Cardiovasculaires (PROTECT)", Inserm, Univ Paris Est Créteil (UPEC), Ecole Nationale Vétérinaire d'Alfort (EnVA), 94700 Maisons-Alfort, France
[5] Faculté de Santé, Université Paris Est Créteil, 94010 Créteil, France
[6] Department of Anaesthesiology and Critical Care Medicine, Amiens University Medical Centre, 80000 Amiens, France
[7] Department of Anaesthesiology and Critical Care Medicine, Dijon University Medical Centre, 21000 Dijon, France
[8] LNC UMR1231, University of Burgundy and Franche-Comté, 21000 Dijon, France
[9] GRC 29, Assistance Publique-Hôpitaux de Paris (AP-HP), Sorbonne Université, 75013 Paris, France
[10] DMU DREAM, Département d'anesthésie et réanimation, Institut de Cardiologie, Hôpital La Pitié-Salpêtrière, 75013 Paris, France
* Correspondence: p.guerci@chru-nancy.fr

Supplementary Table S1. Proposed protocol for LMWH use in the immediate postoperative course of cardiac surgery

	**Coronary artery bypass grafting**	**Heart Valve Replacement**	
	**Aortic root surgery**	Mechanical prosthesis	Bioprosthesis
**Before chest and TEPW removal**	LMWH thromboprophylaxis started 6 to 12 hrs after surgery Ex: Enoxaparin SC 4000UI/day<br><br>+ acetylsalicylic acid 75-100mg/day	LMWH thromboprophylaxis started 6 to 12 hrs after surgery Ex: Enoxaparin SC 4000UI/day<br><br>POD+1: Enoxaparin SC 4000UI x2/day + VKA initiation ➔ to maintain until POD 3 or TEPW removal	LMWH thromboprophylaxis started 6 to 12 hrs after surgery Ex: Enoxaparin SC 4000UI/day<br><br>POD+1 : Enoxaparin SC 4000UI X2/day + VKA initiation<br><br>*N.B.* : for aortic valve, single antiplatelet therapy is possible
After chest and TEPW removal	Enoxaparin SC 4000UI/day + acetylsalicylic acid 75-100mg/day	Therapeutic LMWH dose Enoxaparin 100UI/kg x2/day (max 10 000UI) + VKA until INR is in the desired range Monitor anti-Xa in case of altered renal function (Cl_{creat} < 50 $ml.min^{1}$) or extreme weight (<45kg or >100kg)	Therapeutic LMWH dose Enoxaparin 100UI/kg x2/day (max 10 000UI) + VKA until INR is in the desired range Monitor anti-Xa in case of altered renal function (Cl_{creat} < 50 $ml.min^{1}$) or extreme weight (<45kg or >100kg)

Cl_{creat}: creatinine clearance, LMWH: low molecular weight heparin, POD: postoperative day, SC: subcutaneous, VKA: vitamin K antagonist
